# Acute Drug‐Induced Liver Injury After Four Doses of Nitrofurantoin, as Evaluated for Causality Using the Updated RUCAM, With a Disproportionate Rise in AST—A Novel Case Report

**DOI:** 10.1002/ccr3.73021

**Published:** 2026-06-23

**Authors:** Mahir H. Sampat

**Affiliations:** ^1^ Department of Medicine Renal Medicine, the Royal Wolverhampton NHS Trust Wolverhampton UK

**Keywords:** adverse drug reaction, aspartate transaminase, drug‐induced liver injury, liver diseases, nitrofurantoin, RUCAM

## Abstract

Drug‐induced liver injury remains a severe but underdiscussed adverse effect of nitrofurantoin. This case uniquely describes hepatic injury occurring after four doses of nitrofurantoin, with a disproportionate rise in AST and rapid complete recovery following discontinuation of the drug. A RUCAM score of six supports the probability of DILI.

## 1 Introduction

Urinary tract infections (UTIs) are one of the most common conditions presenting to primary care requiring antibiotics. Prevalance is on the rise, with UTIs causing over 180,000 hospital admissions in England between 2023–2024 and accounting for 1.2 million bed days [1]. Antibiotic use varies based on the type of UTI (lower or upper), severity of presentation, nature and sensitivities of the isolated microorganism and several other patient‐specific factors (sex, past history of UTIs and colonization by microorganisms, immunological status, drug allergies, etc.) Nitrofurantoin is one of the most commonly used antibiotics for the first‐line treatment and prophylaxis of lower UTIs and is effective against a broad range of gram‐positive and gram‐negative bacteria. It has several favorable characteristics including bactericidal action, limited antimicrobial resistance, oral administration, limited effects on bowel flora, and a high cost‐efficacy [2].

Common side effects of nitrofurantoin include nausea/vomiting, gastrointestinal upset, and loss of appetite. Among more severe adverse reactions, pulmonary toxicity is most commonly known and includes a spectrum of presentations from acute pulmonary reactions to more chronic disease and pulmonary fibrosis. Hepatic dysfunction is another serious but less discussed adverse effect and tends to affect more patients with long‐term nitrofurantoin use or pre‐existing hepatic impairment. A new safety advisory was issued by the MHRA in April 2023 alerting prescribers to be vigilant about pulmonary and hepatic adverse drug reactions of nitrofurantoin in adults and children [3].

## 2 Case History

A 52yo female presented to the Emergency Department with a few days' history of non‐specific lower chest/epigastric pain, nausea & vomiting, and an episode of loss of consciousness at home. Past medical history included low mood, anxiety, and low‐grade cervical intraepithelial neoplasia (under surveillance). Her drug history consisted only of propranolol for anxiety, and she declined using any over‐the‐counter or herbal medication, apart from occasional paracetamol (maximum 2 × 500 mg tablets per day) for pain. There was no family history of note. She was a non‐smoker, non‐drinker of alcohol, and declined any recreational drug use.

Vital signs on admission were unremarkable, and the only notable finding on clinical examination was bilateral renal angle tenderness. Examination of cardiovascular, respiratory, and GI systems was unremarkable, as was an ECG. Bloods of note included ALT 74, ALP 138, and Mg 0.52. Inflammatory markers including WBC and CRP were normal, as were U&Es and troponin. Due to her flank tenderness and vomiting, she was empirically treated for a UTI with oral nitrofurantoin and managed conservatively for presumed infective gastroenteritis with fluids, and Mg was appropriately supplemented. She was admitted to the ward due to ongoing pain, vomiting, and poor oral intake requiring intravenous fluids and antiemetics.

## 3 Case History—Investigations, Treatment and Outcome

Blood tests done on the day following admission were remarkable for an acute derangement in liver function—ALT 343, ALP 187 and Bilirubin 39. Additional tests were then requested, which revealed an AST of 2321, Gamma‐GT 577, and Ferritin 2248. Markers of synthetic liver function were unaffected, with a normal INR (1.2), PT (13.5) and albumin (31). There was no clinical change observed, with symptoms the same as on admission. There was no palpable organomegaly, and systemic examination remained unremarkable except for some epigastric tenderness. Nitrofurantoin was the only new drug administered since admission and was stopped while further investigations were carried out. She received a total of 4 doses of 50 mg nitrofurantoin. An extended liver screen was performed which was negative for viral hepatitis (B & C), autoimmune hepatitis and blood borne viruses (HIV, syphilis, EBV and CMV). Liver autoantibodies including anti‐smooth muscle antibody, anti‐mitochondrial antibody, anti‐nuclear antibody and anti‐liver/kidney microsomal antibody were negative, as was anti‐tissue transglutaminase. Serum protein electrophoresis, immunoglobulins, iron studies, paracetamol levels and salicylate levels were also performed and were within normal limits.

An Abdominal Ultrasound scan was performed, which showed some hepatic steatosis, nil else of note. Due to the history of loss of consciousness on admission, transient ischaemic hepatitis was considered as a differential; however, an associated acute kidney injury was not observed, with eGFR and creatinine being normal throughout the admission. Regardless, to exhaust all potential avenues, an NT‐pro‐BNP was checked which was 60, and a transthoracic echocardiogram and 24‐h cardiac monitoring were performed, both of which were unremarkable.

Table 1 displays the trend of laboratory values during the patient's admission. Following cessation of nitrofurantoin, there was a noticeable improvement in transaminases over the next few days with AST dropping to 1218, 458 and 163 from 2321. ALT similarly improved to 282, 200 and 114 from 343, Gamma‐GT was 410 from 577 and ALP 136 from 170. The patient improved clinically, with no further vomiting and well controlled pain, and was discharged after a 7 day hospital stay.

**TABLE 1:** A trend of liver function tests throughout the admission.

Laboratory Values	Day 1	Day 2	Day 3	Day 5	Reference Ranges
Alanine Aminotransferase (ALT)	74	343	282	114	(0–55) IU/L
Aspartate Aminotransferase (AST)	—	2321	1218	163	(5–34) IU/L
GammaGT (GGT)	—	577	515	410	(9–36) U/L
Alkaline Phosphatase (ALP)	138	187	153	136	(30–130) IU/L
Bilirubin	22	39	15	10	(5–26) umol/L
Albumin	33	31	30	34	(30–45) g/L
International Normalized Ratio (INR)	—	1.2	—	1.1	0.8–1.2

## 4 Discussion

Nitrofurantoin‐induced liver injury can range from a mild transaminitis to cholestatic jaundice, fulminant hepatitis, and necrosis. Recovery is often spontaneous but gradual after stopping the drug, with corticosteroids occasionally being used in severe disease albeit without clear evidence or guidelines [4]. There have been a few previous case reports about nitrofurantoin‐induced liver injury; however, these describe prolonged use of the drug. A case report from Southampton (UK) in 2014 described 2 elderly patients admitted with immune‐mediated drug‐induced liver injury (DILI) following nitrofurantoin use; however, the drug was used for 6 and 12 months respectively by these patients. These patients did not have regular monitoring of liver function while taking nitrofurantoin, and therefore these cases had the potential to be recognized earlier or prevented [5]. Another study followed up patients on nitrofurantoin long‐term via DILI registers and found 23 cases of nitrofurantoin‐induced liver injury. Length of use varied from 96 to 760 days and median time to resolution was 81 days. There were no cases of liver transplantation or death; however, 2 patients required immunosuppressive treatment to normalize hepatic enzymes [6]. There was one reported case of a 57 year‐old female requiring a liver transplantation due to nitrofurantoin‐induced liver injury after using nitrofurantoin for 18 months, which demonstrates that while rare, liver injury caused by nitrofurantoin can be severe enough to require a transplant [7].

As per the updated RUCAM scoring for DILI, the R‐factor for this case was calculated as 4.3, indicating mixed hepatic and cholestatic liver injury. The RUCAM score was calculated as six points, indicating that causality of nitrofurantoin for DILI in this case is probable [8].

This case is unusual for several reasons. Most published literature describes hepatic injury from prolonged use of nitrofurantoin; however, this patient only received 4 doses of nitrofurantoin before hepatic injury was observed. One case described acute liver injury with nitrofurantoin; however, this was seen after 12 doses of the drug, and the maximum AST value observed was 717, making this case both more acute in onset and significantly worse in severity [9]. While transaminitis is commonly observed in cases of nitrofurantoin‐induced liver injury, ALT and AST are relatively equally affected. Two cases where AST was higher than ALT; however, the AST:ALT ratio was approximately 1.5–2 in both cases [9, 10]. AST:ALT ratio in this case was 6.8, showing a disproportionately high rise in AST compared to ALT. The cause for this is open to speculation, with a possible explanation being concurrent muscle injury, which has been recognized in conjunction with peripheral neuropathy with nitrofurantoin use. Alcohol is a well‐recognized cause of a raised AST:ALT ratio; however, the patient's history was negative for alcohol use. It is also worth noting that AST is not a factor accounted for on the RUCAM scoring for mixed liver injury.

Finally, as outlined earlier, median time to recovery after nitrofurantoin‐induced liver injury was found to be 81 days; however, a very rapid recovery was observed in this case with liver enzymes normalizing within 1 week, without the use of corticosteroids.

## 5 Conclusion

Drug‐induced liver injury remains a severe but underdiscussed adverse effect of nitrofurantoin. This case uniquely describes hepatic injury occurring after four doses of nitrofurantoin, and rapid complete recovery spontaneously following discontinuation of the drug. A RUCAM score of six supports the probability of nitrofurantoin‐related DILI. While NICE and the BNF recommend monitoring of liver function tests in patients taking nitrofurantoin, this is often not done routinely in day‐to‐day clinical practice. Had this patient received nitrofurantoin in the community, this presentation would not have been diagnosed as early as in hospital, potentially leading to a more severe outcome. An increased awareness from clinicians is required to ensure early recognition and prompt management of liver injury with nitrofurantoin use.

## Author Contributions

**Mahir H. Sampat:** conceptualization, writing – original draft, writing – review and editing, investigation, data curation, methodology.

## Funding

The author has nothing to report.

## Consent

Written informed consent has been obtained from the patient. All information has been sufficiently anonymized. There are no clinical photographs, videos, or patient‐identifying information used.

## Conflicts of Interest

The author declares no conflicts of interest.

## Data Availability

The data that support the findings of this study are available from the corresponding author upon reasonable request.
